# Mendelian randomization and colocalization analysis reveal novel drug targets for myasthenia gravis

**DOI:** 10.1186/s40246-024-00607-7

**Published:** 2024-04-24

**Authors:** Yuzhen Ouyang, Yu Chen, Kangzhi Chen, Zhenwei Tang, Guanzhong Shi, Chunrun Qu, Kaiyue Zhang, Huan Yang

**Affiliations:** 1 Department of Neurology, Xiangya Hospital, Central South University, 410008 Changsha, China; 2 Department of Neurosurgery, Xiangya Hospital, Central South University, 410008 Changsha, China; 3 Department of Dermatology, Second Affiliated Hospital, Zhejiang University School of Medicine, 310013 Hangzhou, China

**Keywords:** Mendelian randomization study, Colocalization analysis, Myasthenia gravis, Drug target, BLyS/APRIL pathway

## Abstract

**Objective:**

Myasthenia gravis (MG) is a complex autoimmune disease affecting the neuromuscular junction with limited drug options, but the field of MG treatment recently benefits from novel biological agents. We performed a drug-targeted Mendelian randomization (MR) study to identify novel therapeutic targets of MG.

**Methods:**

Cis-expression quantitative loci (cis-eQTL), which proxy expression levels for 2176 druggable genes, were used for MR analysis. Causal relationships between genes and disease, identified by eQTL MR analysis, were verified by comprehensive sensitivity, colocalization, and protein quantitative loci (pQTL) MR analyses. The protein-protein interaction (PPI) analysis was also performed to extend targets, followed by enzyme-linked immunosorbent assay (ELISA) to explore the serum level of drug targets in MG patients. A phenome-wide MR analysis was then performed to assess side effects with a clinical trial review assessing druggability.

**Results:**

The eQTL MR analysis has identified eight potential targets for MG, one for early-onset MG and seven for late-onset MG. Further colocalization analyses indicated that CD226, CDC42BPB, PRSS36, and TNFSF12 possess evidence for colocalization with MG or late-onset MG. pQTL MR analyses identified the causal relations of TNFSF12 and CD226 with MG and late-onset MG. Furthermore, PPI analysis has revealed the protein interaction between TNFSF12-TNFSF13(APRIL) and TNFSF12-TNFSF13B(BLyS). Elevated TNFSF13 serum level of MG patients was also identified by ELISA experiments. This study has ultimately proposed three promising therapeutic targets (TNFSF12, TNFSF13, TNFSF13B) of MG.

**Conclusions:**

Three drug targets associated with the BLyS/APRIL pathway have been identified. Multiple biological agents, including telitacicept and belimumab, are promising for MG therapy.

**Supplementary Information:**

The online version contains supplementary material available at 10.1186/s40246-024-00607-7.

## Background

Myasthenia gravis (MG) is a group of autoimmune diseases characterized by fluctuating myasthenia [1]. Multiple antibodies against the acetylcholine receptor (AChR) or other related proteins, such as muscle-specific kinase (MuSK) and lipoprotein receptor-related protein 4 (LRP4) in the postsynaptic membrane of the neuromuscular junction can be detected in the serum of most MG patients, which are applied to classify subgroups (e.g., anti-AChR, anti-MuSK, anti-LRP4, seronegative). MG can also be classified into ocular or generalized MG and early-onset or late-onset MG based on the extent of affected muscles or the age of onset. Given the prevalence of 150 to 250 cases per 1 million and an annual incidence of 8 to 10 cases per 1 million persons, MG and its subgroups constitute significant diseases affecting the neuromuscular junction [2]. Symptomatic therapy such as immunosuppressive therapy, including prednisone or prednisolone with azathioprine, is the cornerstone for MG treatment [3]. With the deepening research on the etiology, increasing therapies, especially biologic agents such as rituximab, are developed with significantly improved therapy efficiency [4]. However, an unmet clinical requirement for better and safer therapy still exists, prompting novel drug target identification.

Bringing novel therapeutic agents from the bench to the bedside is extremely expensive and time-consuming, accompanied by high failure rates [5]. Large-scale randomized clinical trial (RCT) is the classical method to estimate the therapy efficiency of novel drugs. However, the reliance on resources, funding, personnel, and time limits the broad implementation of RCT studies. With the advance of sequencing techniques, omics data, including genomics, transcriptomics, and proteomics, have sprung up in recent years, allowing researchers to identify novel drug targets from multi-omics data. The amount of genome-wide association study (GWAS) data grows significantly, which is suitable for research on neurological diseases because of the close correlation between these diseases and complex hereditary backgrounds.

The Mendelian randomization (MR) study is an alternative method to explore the potential causal relationship between exposure and outcome based on the GWAS data [6]. And the drug-targeted MR analysis commonly adopts cis-expression quantitative trait loci (eQTL) or protein quantitative trait loci (pQTL) data to proxy the gene expression level. Such MR analysis has been applied to multiple diseases, including COVID-19, Parkinson’s disease, and aortic aneurysms [7–9]. MR analyses also propose potential therapeutic targets for multiple sclerosis (MS) and numerous neurodegenerative diseases [10, 11]. A recent protein-centric omics integration analysis also explored potential therapeutic targets for 10 autoimmune diseases including MG [12]. Proteome-wide association (PWAS), MR, and colocalization analyses identify PRSS8 and CTSH as promising drug targets for MG. However, no drugs targeting these two targets are conducting clinical trials for MG or similar rheumatic diseases. Therefore, we decided to apply multi-omics data on RNA and protein levels with enzyme-linked immunosorbent assay (ELISA) on patients’ serum samples to explore more promising drugs for MG.

Given the dilemma of increasing demands for drug innovation of MG and limited research progress, we aimed to perform a drug-targeted MR analysis based on the eQTL data of druggable targets, serum pQTL data, and GWAS data of AChR antibody-positive MG. Colocalization and comprehensive sensitivity analyses were conducted to exclude potential bias, including heterogeneity, pleiotropy, and directionality analyses. Finally, protein-protein interaction (PPI) analysis, ELISA, current clinical trial information review, and evaluation of potential side effects were also performed.

## Methods

### Design of research

**Fig. 1:**
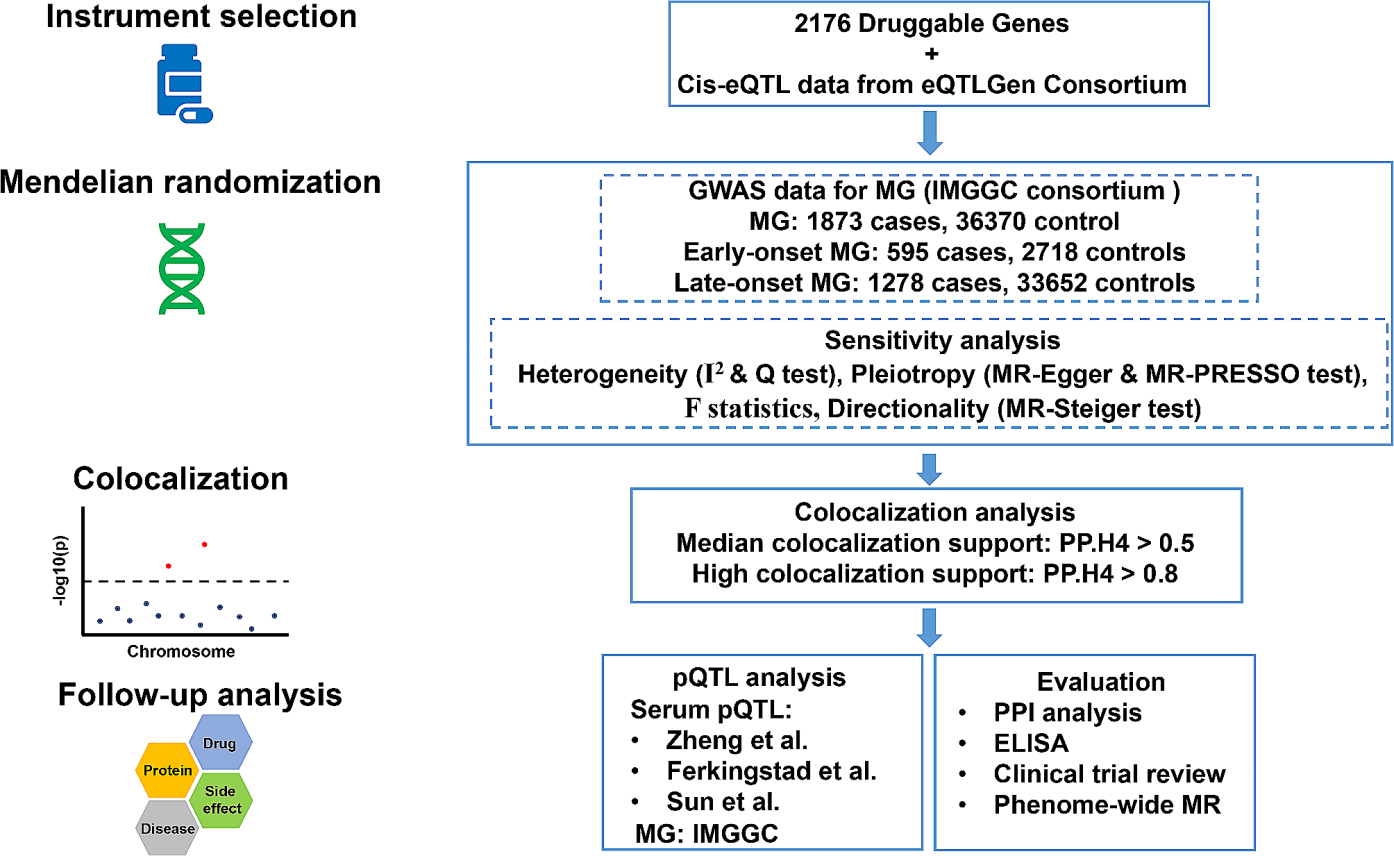
Overview of the study design. Promising drug targets are identified by multiple steps including eQTL MR, sensitivity, colocalization, pQTL MR analysis, ELISA, clinical trial review, and safety evaluation. eQTL, expression quantitative trait loci; pQTL, protein quantitative trait loci; MR, mendelian randomization; MG, myasthenia gravis; IMGGC, International Myasthenia Gravis Genomics Consortium; PPI, protein-protein interaction

Our study aimed to discover potential therapeutic targets for MG by drug-targeted MR analyses. Figure 1 displays a study flowchart. The druggable genome was first linked to the cis-eQTL data to simulate the expression level of each gene. Second, potential causal correlations between the expression level of druggable genes and the risk of MG with early-onset and late-onset subgroups were explored by two-sample MR analysis. Further sensitivity and colocalization analyses were performed to rule out any possible bias. pQTL MR analyses of promising therapeutic targets were subsequently conducted for validation with the PPI analysis to identify more potential drug targets and related signaling pathways. ELISA experiments were performed to identify the serum level of potential drug targets in MG patients. Finally, the clinical trial of each target was evaluated, and possible side effects were explored by the Phenome-wide MR (Phe-MR) analysis.

### Data resource

A total of 4479 protein-coding genes were identified to be drugged or druggable, which was comprised of three tiers of drugs: tier 1 (Targets of approved drugs or drugs in clinical development), tier 2 (Proteins related to drug targets or drug-like compounds), and tier 3 (Members of critical drug target families and extracellular proteins) [13]. The statistically significant cis-eQTL (false discovery rate (FDR) < 0.05) from the eQTL consortium and meta-analysis of the peripheral blood (31,684 mostly European-ancestry individuals) was adopted to proxy druggable genes [14]. Variants located within the major histocompatibility complex region (chromosome 6: 26–34 Mb) were removed because of the linkage disequilibrium structure in this region. Cis-eQTL loci within ± 100 kb from each gene’s position were selected with 2176 druggable genes of available cis-eQTL blood in the final.

GWAS statistic of acetylcholine receptor antibody-positive MG was obtained from the research of the International Myasthenia Gravis Genomics Consortium (IMGGC) with subgroup data of patients with early- or late-onset MG [15]. Information such as the ethical approval can be found in Table 1 and the original article.

**Table 1:** Characteristics of included GWAS data

Trait	Consortium	Sample size	Population	Journal	Year
Druggable Genes	/	4479 genes	European	Science Translational Medicine	2017
eQTL					
Blood	eQTLGen	31,684 individuals	European	Nature Genetics	2021
pQTL					
Plasma proteome Plasma proteome Plasma proteome	deCODE / Pharma Proteomics	4,907 proteins 1,699 proteins 2,923 proteins	European European European	Nature Genetics Nature Genetics Nature	2021 2020 2023
Disease					
Myasthenia Gravis (MG) Early-onset MG Late-onset MG	IMGGC IMGGC IMGGC	38,243 (1873 cases, 36,370 controls) 3,313 (595 cases, 2,718 controls) 34,930 (1,278 cases, 33,652 controls)	European European European	PNAS PNAS PNAS	2022 2022 2022

### MR analysis

Candidate instrument variables (IVs) were selected at a significance level of *P* < 5e-8. The parameters of the clump function in the “TwosampleMR” R package (0.5.6) were set as r^2^ = 0.1, kb = 10,000 (EUR population) to guarantee the independence of IVs, reducing the impact of linkage disequilibrium. Palindromic SNPs were excluded before the MR analysis.

In the principal MR analysis, the Wald ratio method was applied to calculate unconfounded MR estimates of each single nucleotide polymorphism (SNPs), and the estimation was meta-analyzed by the Inverse variance weighted (IVW), MR-Egger, weighted mode, and weighted median models. Bonferroni corrections were applied to adjust the significance thresholds for the analysis. In the analysis, the *p*-value below 2.3e-5 (0.05/2176) was considered significant. While the IVW functioned as the primary test, results from the other four tests provided the supplement.

### Sensitivity analysis

The F-statistic of each gene was first calculated to identify whether weak-instrument bias existed. IVW method assumes the SNP should affect outcome only through exposure, which will be violated if pleiotropy exists [16]. The MR-Egger method can be applied in the existence of the pleiotropy effect. The pleiotropy effect is suggested if the y-intercept deviates from zero significantly [17]. Additionally, the MR pleiotropy residual sum and outlier (MR-PRESSO) test (“MRPRESSO” R package, 1.0) was also conducted to explore the potential horizontal pleiotropy [18]. Q tests of IVW and MR-Egger, and I^2^ tests were performed to exclude the heterogeneity of results. In addition, the MR Steiger directionality test was adopted to explore the robustness of the causality direction.

### Colocalization analysis

Subsequent colocalization analysis was performed for MG risk of significant MR results in MR analyses using the R package “coloc” (5.1.0.1) with default priors (p1 = 10^− 4^, p2 = 10^− 4^, p12 = 10^− 5^, p1, p2, and p12 are the prior possibilities that an SNP is significantly associated with the expression level of a gene, the disease outcome, or both, respectively) [19]. The colocalization provides the posterior possibilities of five hypotheses (H0: no association with the gene and diseases; H1: association with the expression level of the gene but not the disease; H2: association with the disease but not the gene expression; H3: association with both the gene and expression level with a different causal variant; H4: association with both the gene and expression level with the same causal variant) [19]. And results were visualized by the LocusZoom (http://locuszoom.org/) [20].

### pQTL analysis

The primary analysis of plasma pQTL was conducted based on the pQTL data from Ferkingstad et al. (4,907 plasma proteins from 35,559 participants) [21]. The pQTL data reported by Zheng et al. [22], including five published pQTL GWASs, and Sun et al. [23], were used for extension. pQTL data of promising drug targets identified in previous eQTL analyses were retrieved and downloaded. pQTL meets the following standards are included: (1) Genome-wide significance (*p* < 5*10^− 8^); (2) Without linkage disequilibrium (clump: r^2^ = 0.1, kb = 10,000); (3) cis- and trans-acting pQTL; (4) Located outside the major histocompatibility complex (MHC) region (chr6, 26-34 Mb). The MR and sensitivity analysis pipeline was identical to the previous eQTL analysis. The threshold *p*-value of significance was set as 0.05.

### Protein-protein interaction analysis

PPI analysis aimed to explore the interactions among the prioritized drug targets and other proteins, especially targets for medications already on the market. All PPI analyses were conducted using the Search Tool for the Retrieval of Interacting Genes (STRING) database version 12.0 (https://string-db.org.) with the minimum required interaction score of 0.4.

### ELISA

Whole blood samples from 8 AChR antibody-positive MG patients and 8 healthy controls were centrifugated (1000 g, 10 min), and the supernatant was saved in a centrifuge tube. The serum protein level was detected by ELISA kits (mlbio, China). The protein in the serum was first captured by the antibody immobilized on the plate, and the biotin-labeled antibody was then added to bind to the protein, incubating for 30 min. Unbound proteins and antibodies were washed away, and horseradish peroxidase (HRP)-labeled antibodies were added to incubate for 30 min. At the end of incubation, unbound antibodies were removed by a washing step. The substrate solution was added and incubated for 15 min, then the stop solution was added and the protein level was measured by an optical density of 450 nm within 15 min. Results were analyzed by unpaired t-test with Welch’s correction.

### Assessment of druggability and safety

Each prioritized target’s clinical trials were reviewed on the Open Targets platform (https://platform.opentargets.org/). The druggability of each potential drug target was evaluated and summarized. Finally, the Phe-MR analysis was conducted to assess the safety based on the on-target side effects. Phenotypes, including 821 traits, were retrieved from the Medical Research Council Integrative Epidemiology Unit (MRC IEU) OpenGWAS [10, 24, 25]. The selection criteria were the same as a previous Phe-MR analysis: (1) Disease, disease-related clinical manifestations, and disease-related risk factors based on middle-aged or older adults. (2) Sample size larger than 10,000. (3) European or mixed ethnicity [10]. Subsequent Phe-MR analysis was performed to identify potential side effects of each promising drug target with Bonferroni correction based on the pQTL data.

## Results

### eQTL MR analysis identifies 13 drug targets for MG

**Fig. 2:**
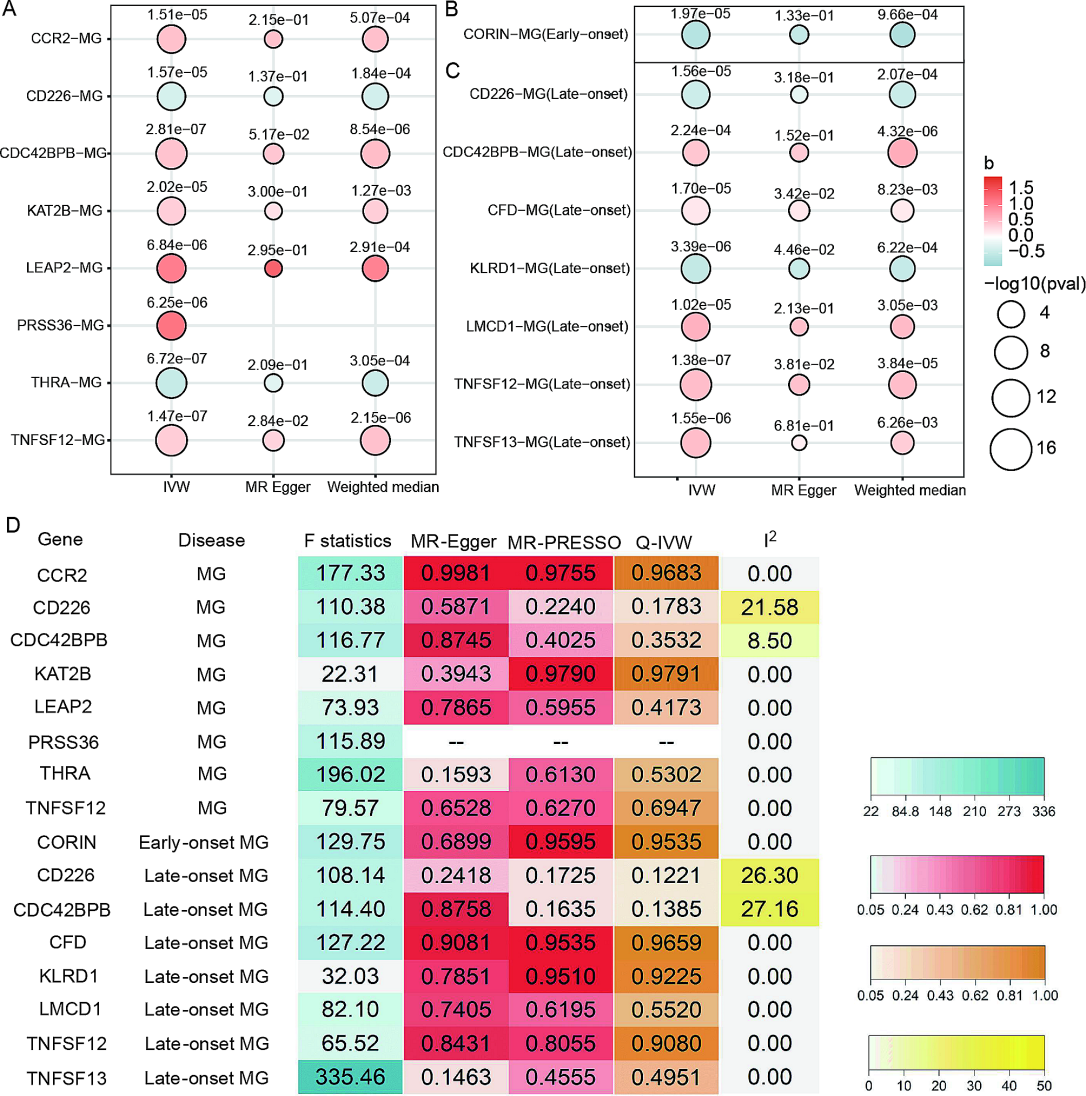
eQTL MR results of each gene-disease association. (**A-C**) MR results of druggable genes with (**A**) MG, (**B**) Early-onset MG, and (**C**) Late-onset MG. Circle size indicates the *p*-value and the color of circles suggests the beta value of MR results of each gene-disease pair. (**D**) Sensitivity analysis result of 16 gene-disease associations

A dataset including 2176 druggable genes comprised of three tiers of genes encoding drug targets was utilized for discovering novel drug targets of MG [13]. After eQTL proxy, clump, and harmonization, genetically proxied expression of 8 genes (CCR2, CD226, TNFSF12, CDC42BPB, KAT2B, LEAP2, PRSS36, THRA), 1 gene (CORIN), and 7 genes (CCR2, CDC42BPB, TNFSF12, TNFSF13, CFD, KLRD1, LMCD1) were significantly associated with the risk of MG, early-onset MG, and late-onset MG, respectively. These 16 significant gene-disease correlations involving 13 promising targets are exhibited in Fig. 2A-C. Among these genes, IVW results of CDC42BPB-Late onset MG didn’t pass the Bonferroni correction, but the *p*-value of the weighted median method was smaller than 2.3e-5. This gene was first kept for further analysis to explore whether they were potential drug targets. The primary MR results of MG were summarized in Table S1. In addition, IVs of each significant gene were summarized in Table S2.

### MR quality control to guarantee the robustness of MR results

Quality control steps were conducted to filter out drug targets with robustness. The Wald ratio method calculated the MR effect of each SNP, which was meta-analyzed by multiple meta-analysis methods of MR effects, including IVW, MR-Egger, Weighted median, and Weighted mode. The predicted directions of these 13 genes were robust because different meta-analysis methods yielded the same predicted direction. Besides, MR-Egger intercept and MR-PRESSO tests were performed to identify potential pleiotropy, summarized in Table S3. The pleiotropy bias did not exist based on the results of MR-Egger and MR-PRESSO. In addition, Cochran’s Q and I^2^ tests in Table S4 were performed in genes that have passed the pleiotropy test to evaluate the heterogeneity. The heterogeneity did not exist in the 13 genes referring to the results of Cochran’s Q (*p* < 0.05) and I^2^ tests (I^2^ > 50). All genes passed MR-steiger tests in Table S5, guaranteeing the direction of causal relationship from gene expression levels to diseases. The F statistics, I^2^ results, and *p*-value of MR-Egger intercept, MR-PRESSO, and Q-IVW of these gene-disease pairs are exhibited in Fig. 2D.

After these sensitivity analyses, 8 genes (CCR2, CD226, THRA, KAT2B, LEAP2, PRSS36, CDC42BPB, TNFSF12) with MG, 1 gene (CORIN) with early-onset MG, 7 genes (CD226, CDC42BPB, CFD, KLRD1, LMCD1, TNFSF12, TNFSF13) with late-onset MG were retained as potential therapeutic targets.

### Colocalization results

**Fig. 3:**
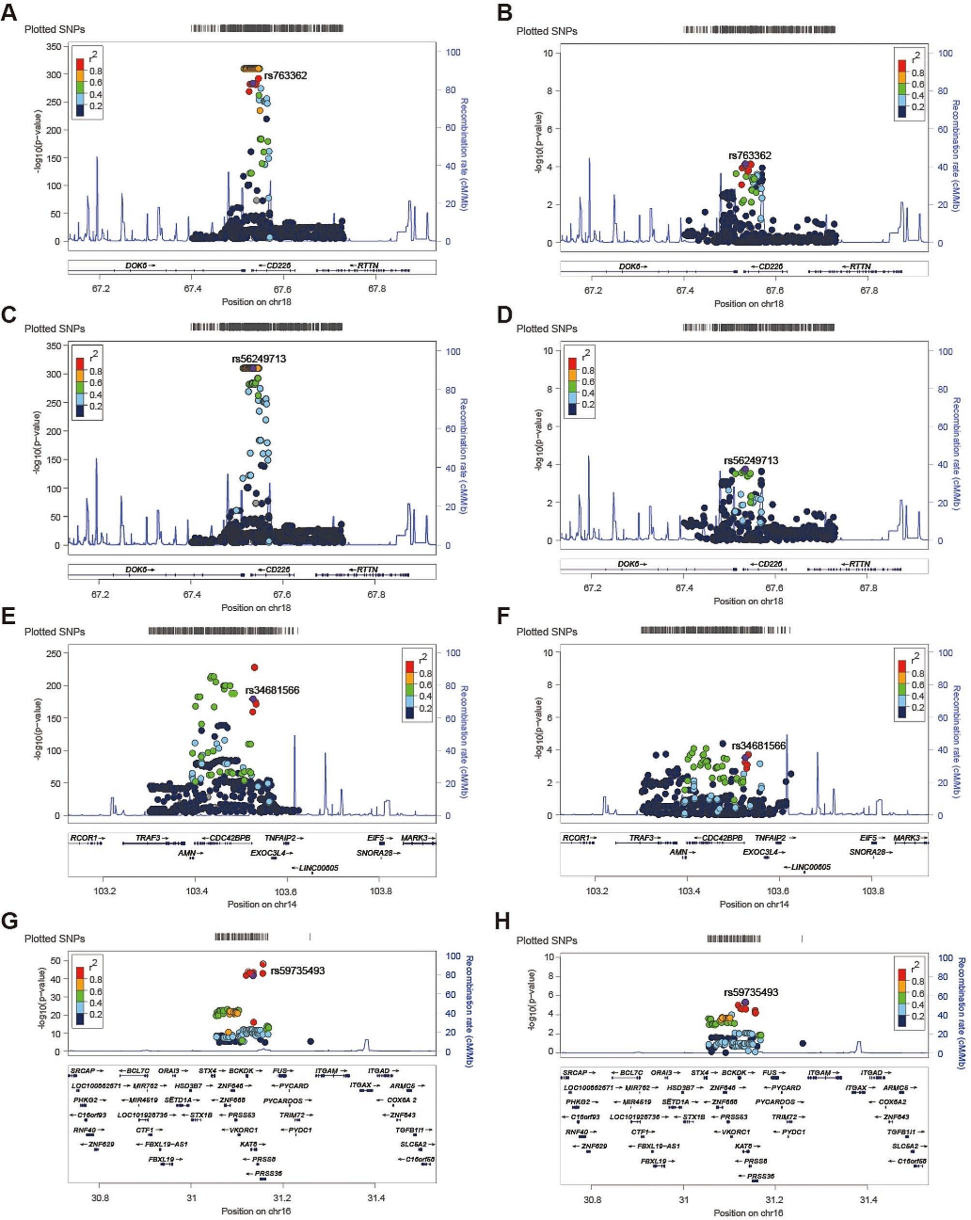
Regional Manhattan plot of associations of SNPs with CD226, CDC42BPB, and PRSS36. (**A**) rs763362 is used to proxy the serum level of CD226. (**B**) rs763362 with its flanking 400 kb region in MG. (**C**) rs56249713 is used to proxy the serum level of CD226. (**D**) rs56249713 with its flanking 400 kb region in late-onset MG. (**E**) rs34681566 is utilized to proxy the serum level of CDC42BPB. (**F**) rs34681566 with its flanking 400 kb region in MG. (**G**) rs59735493 is utilized to proxy the serum level of PRSS36. (**H**) rs59735493 with its flanking region in MG

The close linkage disequilibrium also leads to inaccurate MR results because of the horizontal pleiotropy, where the associations of SNP-exposure and SNP-outcome fall on distinct causal SNPs [26]. Colocalization can assist in evaluating whether a single SNP is significantly associated with both the exposure and outcome simultaneously [19]. The posterior probability of hypothesis 4 (PP.H4) of at least 80% suggests highly likely to colocalize, and the PP.H4 of at least 50% indicates likely to colocalize [10, 27, 28]. The colocalization analyses were performed, and the results are summarized in Table S6. The results suggested that CD226 was identified as a candidate for the risk of MG (PP.H4 = 0.86, Fig. 3A-B) and late-onset MG (PP.H4 = 0.93, Fig. 3C-D). Besides, CDC42BPB and MG (PP.H4 = 0.95, Fig. 3E-F), PRSS36 and MG (PP.H4 = 0.92, Fig. 3G-H) likely shared a causal variant. Besides, the PP.H4 of CDC42BPB with late-onset MG exceeded 0.5, and PP.H3 + PP.H4 of TNFSF12 with MG was larger than 0.8, indicating the bias of these two genes was also acceptable.

### Protein-level drug target identification

**Fig. 4:**
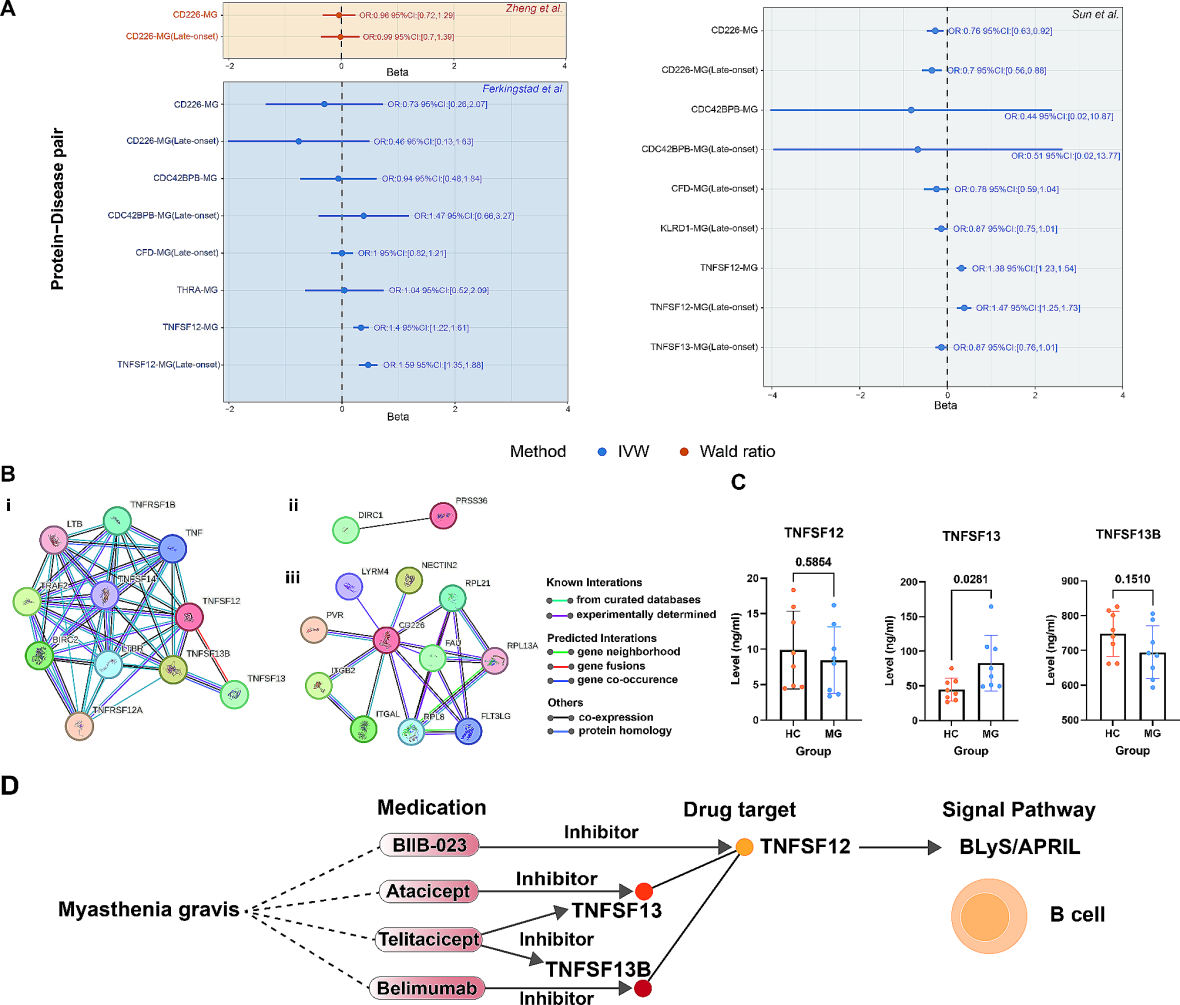
Protein-level drug target identification. (**A**) The forest plot exhibits MR results of all protein-disease pairs with available pQTL data. The center of the error bars represents the odds ratio of MG, and per 1-standard-deviation increase in protein levels, which is calculated by Wald ratio (1 SNP) or IVW (> 1 SNP). MR analyses are based on three pQTL datasets (Zheng et al., Ferkingstad et al., Sun et al.), which can be distinguished by background color. (**B**) Protein-protein interaction (PPI) results of (i) TNFSF12, (ii) PRSS36, and (iii) CD226. (**C**) Serum levels of TNFSF12, TNFSF13, and TNFSF13B detected by ELISA experiments. (**D**) Schematic illustration of promising drug targets with its targeted medications and signal pathways. PPI, protein-protein interaction; ELISA, enzyme-linked immunosorbent assay; OR, odds ratio; 95% CI, 95% confidential interval **Supporting information**

Compared with the expression level, the protein level (protein quantitative loci, pQTL) can mimic the drug effect more effectively. However, the GWAS research of protein was limited, and GWAS data from three studies of plasma proteome (Zheng et al., Ferkingstad et al., Sun et al.) were used for pQTL analysis [21–23]. Among all pQTL MR results in Fig. 4A, associations of TNFSF12 with MG and late-onset MG were significant based on two datasets (Ferkingstad et al., Sun et al.). Besides, the pQTL analysis based on data from Sun et al. reported significant associations of CD226 with MG and late-onset MG. All pQTL MR results are summarized in Table S7. All supporting evidence of 12 druggable genes has been summarized in Table 2. TNFSF12 with its related pathways is a promising drug target with evidence from eQTL, colocalization, and pQTL levels. While CD226 passed the pQTL test in one dataset (Sun et al.), the lack of pQTL data restricted the verification of PRSS36 on the protein level.

**Table 2:** Supporting evidence of druggable genes

Gene	Disease	eQTL	Heterogeneity	Pleiotropy	Direction	Colocalization	pQTL
CDC42BPB	MG MG (Late-onset)	*√* *√*	*√* *√*	*√* *√*	*√* *√*	*√* *√*	*×* *×*
CD226	MG MG (Late-onset)	*√* *√*	*√* *√*	*√* *√*	*√* *√*	*√* *√*	*×/√* *×/√*
TNFSF12	MG MG (Late-onset)	*√* *√*	*√* *√*	*√* *√*	*√* *√*	*√* *√*	*√* *√*
KAT2B	MG	*√*	*√*	*√*	*√*	*×*	
LEAP2	MG	*√*	*√*	*√*	*√*	*×*	
THRA	MG	*√*	*√*	*√*	*√*	*×*	*×*
PRSS36	MG	*√*	*√*		*√*	*√*	
CORIN	MG (Early-onset)	*√*	*√*	*√*	*√*	*×*	
TNFSF13	MG (Late-onset)	*√*	*√*	*√*	*√*	*×*	*×*
CFD	MG (Late-onset)	*√*	*√*	*√*	*√*	*×*	*×*
KLRD1	MG (Late-onset)	*√*	*√*	*√*	*√*	*×*	*×*
LMCD1	MG (Late-onset)	*√*	*√*	*√*	*√*	*×*	

*√* pass, *×* fail, *blank* not possible to test, *×/√* pass in some datasets, *eQTL* expression quantitative trait locus, *pQTL* protein quantitative trait locus

Additional supporting information can be found online at the end of the article

PPI analyses were subsequently conducted to reveal interactions between prioritized proteins (TNFSF12, CD226, and PRSS36) and other proteins, especially druggable targets. Based on the STRING database, TNFSF12-TNFSF13 (APRIL) and TNFSF12-TNFSF13B (BLyS) were considered the most reliable interactions (known interactions) in Fig. 4B. The PPI analysis also identified the co-expression correlation of TNFSF12-TNFSF13. In addition, as shown in Table S7, the significant association of TNFSF13 with MG and early-onset MG was also identified in pQTL MR analysis. Drugs targeting BLyS/APRIL, such as belimumab and telitacicept, are promising for treating MG patients. As for the PRSS36, the co-expression relationship of PRSS36-DIRC1 was identified. A previous study also proposed PRSS8 as a potential drug target for MG [12]. The serine protease pathway deserved further attention for MG therapy. CD226 was previously reported to be associated with multiple autoimmune diseases such as systemic lupus erythematosus (SLE) [29]. However, no existing drugs targeting PRSS36, DIRC1, CD226 and its interacted proteins have been reported. Therefore, TNFSF12, TNFSF13, and TNFSF13B are more likely to be promising drug targets for MG.

ELISA was subsequently conducted to ascertain whether the serum levels of TNFSF12, TNFSF13, and TNFSF13B were significantly different between MG patients and healthy controls. The characteristics of MG patients and health controls have been summarized in Table S8. As shown in Fig. 4C, while the TNFSF12 or TNFSF13B level difference was not significant, the serum level of TNFSF13 in MG patients significantly increased compared with that in healthy control. Therefore, the TNFSF13(APRIL)/TNFSF13B(BLyS) pathway was considered a promising target for MG therapy. Furthermore, the promising drug targets with their targeted medications and related signaling pathways are finally exhibited in Fig. 4D. Novel biological agents such as telitacicept possess the potential to improve the therapeutic outcome of MG patients.

### Druggability and safety

A systematic review of filtered drug targets was also conducted using the Open Targets platform. Clinical trials related to 3 drug targets (TNFSF12, TNFSF13, TNFSF13B) are presented in Table S9. The clinical trial of TNFSF12 inhibitor (BIIB-023 and RO-5,458,640) stagnated in phases 1 and 2, which was far from clinical application. The therapeutic prospects of TNFSF13 (APRIL) inhibitor (atacicept) on autoimmune diseases such as lupus nephritis are huge as the clinical trials have proceeded to phase 3. Moreover, the TNFSF13B (BLyS) inhibitor, such as the belimumab, has passed the clinical trials and has been approved for SLE therapy. Clinical trials of other TNFSF13B inhibitors (e.g., blisibimod, tabalumab) also advanced to phase 3. Another dual-target (BLyS + APRIL) inhibitor, telitacicept, has completed a phase 2 trial of MG therapy. Clinical trials of telitacicept in other indications such as SLE and primary Sjogren’s syndrome were also performed [30, 31]. These results suggested that biological agents against BLyS/APRIL pathways were promising for treating autoimmune diseases.

The Phe-MR analysis explored Potential side effects on 3 targets (TNFSF12, TNFSF13, TNFSF13B). 821 phenotypes in Table S10 were included for Phe-MR analysis. While TNFSF13B exhibited negligible side effects, the potential side effects of TNFSF12 and TNFSF13 deserved attention. The TNFSF13 level was associated with numerous serum lipid substances such as lipoprotein and cholesterol. The negative correlation between the serum TNFSF12 level and circulatory system diseases such as atrial fibrillation and stroke suggested the probable side effects of TNFSF12 inhibitors, which deserved extra attention in circulatory disorders. The positive correlation between the expression level of TNFSF12 and rheumatoid arthritis (RA) or hypertension indicated the possible extension of TNFSF12 indications. In general, the safety of each drug target has been preliminarily evaluated by the Phe-MR analysis with significant results summarized in Table S11, but further evaluation should be conducted by experiments on animal models and clinical trials.

## Discussion

This research explicitly sought to identify promising drug targets for MG. eQTL MR analyses prioritized 13 drug targets: 8 genes for MG, 1 gene for early-onset MG, and 7 genes for late-onset MG. Comprehensive sensitivity analyses, including heterogeneity, pleiotropy, and directionality tests, found no potential bias. Colocalization analyses supported CD226, CDC42BPB, PRSS36, and TNFSF12 as promising targets for MG. pQTL MR analyses revealed significant causal protein-disease relations of TNFSF12 and CD226 with MG and late-onset MG. Further PPI analyses proposed the protein interaction of TNFSF12-TNFSF13 (APRIL), and TNFSF12-TNFSF13B (BLyS), suggesting 3 promising drug targets (TNFSF12, TNFSF13, TNFSF13B). Subsequent ELISA experiments identified elevated TNFSF13 serum levels in MG patients. The importance of BLyS/APRIL and other promising signaling pathways for MG-targeted therapy is discussed hereinafter.

Though most patients with MG can maintain a near-normal quality of life, the therapy efficiency is still insufficient. Numerous problems, such as adverse effects, long course of treatment, and refractory MG, still exist in the current MG therapy. The MG Foundation of America post-interventional status (MGFA-PIS) of drug refractory MG patients has not improved after drug administration [32]. Initial deterioration, even myasthenic crisis, can be observed in some MG patients receiving intravenous methylprednisolone therapy [33]. Therefore, novel drugs are needed, of which newer biological agents are promising [34].

The protein interaction of TNFSF12-TNFSF13 (APRIL) and TNFSF12-TNFSF13B (BLyS) indicated the BLyS/APRIL pathway is an important therapeutic target for MG. The BLyS/APRIL pathway includes two cytokines, BLyS and APRIL, and their three receptors, transmembrane activator and CAML interactor (TACI), B-cell maturation antigen (BCMA), and B-cell activation factor receptor (BAFFR), which is crucial for B cell maintenance [35]. Our research provides compelling evidence for drugs targeting the BLyS/APRIL pathway such as telitacicept. Telitacicept has been granted orphan drug status of MG by the Food and Drug Administration (FDA) and received its first approval for treating SLE in China [36]. Our previous research has also reported successful therapy outcomes of telitacicept on neurofascin-155 (NF155) + autoimmune nodopathy [37]. Further clinical trials of telitacicept on MG are necessary, and a phase 3 clinical trial of telitacicept for generalized AChR + MG therapy is being conducted by our research group. Clinical trials of telitacicept in other diseases such as primary Sjogren’s syndrome and SLE were also performed [30, 31]. Besides, atacicept is a fusion protein targeting BLyS and APRIL, which consists of fragment crystallizable (Fc) regions of human IgG1 linked to the extracellular binding domain of TACI. The successful application of atacicept in treating SLE has been reported previously [38]. However, the clinical trial of atacicept in multiple sclerosis (ATAMS) failed because of increased clinical disease activity associated with atacicept, which was probably related to BCMA function during inflammation [39, 40]. The therapeutic efficiency of another drug, belimumab, targeting the TNFSF13B, has been explored in the phase 4 clinical trial of SLE [41, 42]. Belimumab has been considered a critical disease modifier in the SLE treatment paradigms [43]. Besides, a phase-II study (NCT01480596) of belimumab therapy for patients with generalized myasthenia gravis reported the improvement of quantitative myasthenia gravis (QMG) score in the belimumab group but without statistically significant difference compared with that of the placebo group [44]. Further research on belimumab for MG therapy was still required. Other TNFSF13B-targeted drugs (e.g., tabalumab, tibulizumab, blisibimod) are also worthy of attention. The importance of the BLyS/APRIL pathway is self-evident, and telitacicept was worthy of further clinical trials in MG, and promising as an MG immunotherapy choice in the future.

TNFSF12 (TWEAK) itself is a multifunctional cytokine belonging to the TNF superfamily, which can bind to a cell surface receptor named Fn14 (TNFRSF12A), triggering cellular responses [45]. The TWEAK/Fn14 axis is implicated in the pathophysiology of several diseases, such as inflammatory bowel and neurological diseases [46]. Besides, TWEAK- or Fn14-targeted agents, including monoclonal antibodies (BIIB-023), fusion proteins, and immunotoxins, were developed for the therapy of RA and lupus nephritis (NCT00771329, NCT01499355, NCT01930890). In addition, the severity of experimental autoimmune encephalomyelitis (EAE) and immune cell infiltration was reduced after the anti-TWEAK antibody usage [47]. The Fn14-TRAIL fusion protein, which can inhibit TWEAK/Fn14 signaling and stimulate TRAIL/TRAILR signaling, attenuated the severity of EAE [48]. Our research proposed TNFSF12 as a potential drug target for MG. Therefore, the clinical and economic value of TNFSF12 was probably underestimated.

In addition to TNFSF12, TNFSF13, and TNFSF13B, the complement system and neonatal Fc receptor (FcRn) also attract attention in MG therapy. For example, a C5 inhibitor, eculizumab, succeeded in anti-AChR-positive refractory generalized MG (gMG) treatment [49]. Eculizumab was associated with a more frequent minimal manifestation of MG and better clinical outcomes than rituximab [50]. Moreover, zilucoplan and ravulizumab were also successfully applied for generalized MG treatment [51, 52]. Furthermore, FcRn antagonists such as efgartigimod and batoclimab exhibited satisfactory therapeutic outcomes for MG patients [53, 54]. In general, novel drugs blocking B cell activation, the complement system, and FcRn served as promising choices to solve therapeutic dilemmas in the immunotherapy era of MG. These biological agents can greatly improve treatment response for MG patients in addition to classical glucocorticoids and immunosuppressants.

A previous study has proposed CTSH and PRSS8 as promising drug targets of MG by PWAS, MR, and colocalization analysis [12]. Based on druggable eQTL gene-colocalization-pQTL-PPI research strategy and larger pQTL datasets, our research proposed more potential targets (TNFSF12, PRSS36, CD226, TNFSF13, TNFSF13B). Subsequent ELISA on patients’ samples verified the elevated serum level of TNFSF13 in MG. We finally proposed TNFSF13 and TNFSF13B as the most promising drug targets, indicating the significance of the BLyS/APRIL pathway of MG treatment. This also emphasizes that the B cell activation blockade therapy is promising to be added to the existing treatment paradigm of MG, which can inspire other similar autoimmune disease treatments. BLyS/APRIL blockade by telitacicept possesses great therapeutic potential, especially for drug-refractory MG patients. Therefore, our research results are informative, robust, and meaningful.

Several limitations should be addressed in this study. First, MR can’t recapitulate clinical trials, which can only provide clues for promising therapeutic targets. The MR analysis is based on prerequisites including lifelong, long-dose administration of drugs, which is different from relatively high-dose and short-time clinical drug usage. Second, subgroups influence therapeutic options and the prognosis of MG. As the GWAS data we used only includes the AChR antibody-positive patients with early- and late-onset subgroups, the generalization of proposed promising targets to other subgroups (e.g., anti-MuSK, anti-LRP4, seronegative) requires further studies. The sample size of our ELISA experiments also restricted further subgroup analysis. Third, while eQTL data consisted of expression levels of genes in peripheral blood mononuclear cells, the pQTL data was generated from detecting serum protein levels. This discrepancy was related to the failed verification of several targets due to the lack of data, which probably led to missing some therapeutic targets. Finally, clinical trials of BLyS/APRIL pathway by telitacicept or other drugs are necessary to evaluate their clinical treatment efficiency. The immune-horizon exploration after telitacicept treatment of MG patients is also crucial to understanding the drug efficiency, ascertaining therapeutic dilemmas, and realizing further improvement. Besides, as B cell depletion therapy by rituximab and B cell activation blockade treatment are prosperous, more innovative therapies targeting B cells can be explored through mechanistic pathway investigation and in-vivo experimental autoimmune myasthenia gravis (EAMG) models.

## Conclusion

In conclusion, this study proposes five potential drug targets (TNFSF12, PRSS36, CD226, TNFSF13, and TNFSF13B) for MG through eQTL, colocalization, pQTL, and PPI analyses. The elevated serum level of TNFSF13 in MG patients was detected by ELISA experiments. TNFSF12, TNFSF13(APRIL), and TNFSF13B (BLyS) emerge as the most robust therapeutic candidates with multiple available drugs, including telitacicept, atacicept, and belimumab. BLyS/APRIL signaling is a promising pathway for MG therapy in the era of biologics. Further successful clinical trials of these targets in MG are necessary to provide valuable insights for drug repurposing endeavors.

### Electronic supplementary material

Below is the link to the electronic supplementary material.

**Supplementary Material 1**: **Table S1.** Significant results of eQTL MR analysis. **Table S2.** Genetic information of SNPs associated with druggable genes. **Table S3.** Results of MR-Egger and MR-PRESSO analysis for pleiotropy test. **Table S4.** Results of Q and I^2^ analysis for heterogeneity test. **Table S5.** Results of F statistics and Steiger filtering analysis. **Table S6.** Results of colocalization analysis. **Table S7.** Results of pQTL MR analysis. **Table S8.** Characteristics of MG patients and healthy controls. **Table S9.** Clinical trial information of promising drug targets. **Table S10.** 821 phenotypes included for Phenome-wide MR (Phe-MR) analysis. **Table S11.** Results of Phe-MR analysis.

## Data Availability

No datasets were generated or analysed during the current study.

## Abbreviations

MG: Myasthenia gravis
AChR: Acetylcholine receptor
MuSK: Muscle-specific kinase
LRP4: Lipoprotein receptor-related protein 4
RCT: Randomized clinical trial
GWAS: Genome-wide association study
MR: Mendelian randomization
eQTL: Expression quantitative trait loci
pQTL: Protein quantitative trait loci
MS: Multiple sclerosis
PWAS: Proteome-wide association
ELISA: Enzyme-linked immunosorbent assay
PPI: Protein-protein interaction
Phe-MR: Phenome-wide MR
FDR: False discovery rate
IV: Instrumental variable
IVW: Inverse variance weighted
SNP: Single nucleotide polymorphisms
MR-PRESSO: MR pleiotropy residual sum and outlier
PP.H4: Posterior probability of hypothesis 4
OR: Odds ratio
MHC: Major histocompatibility complex
STRING: Search Tool for the Retrieval of Interacting Genes
HRP: Horseradish peroxidase
95% CI: 95% confidential interval
RA: Rheumatoid arthritis
BLyS: B-lymphocyte stimulator
APRIL: A proliferation-inducing ligand
SLE: Systemic lupus erythematosus
TACI: Transmembrane activator and CAML interactor
BCMA: B-cell maturation antigen
BAFFR: B-cell activation factor receptor
FDA: Food and Drug Administration
Fc: Fragment crystallizable
QMG: Quantitative myasthenia gravis
EAE: Experimental autoimmune encephalomyelitis
FcRn: Neonatal Fc receptor
gMG: Generalized myasthenia gravis
EAMG: Experimental autoimmune myasthenia gravis
